# Particle Physics in High School: A Diagnose Study

**DOI:** 10.1371/journal.pone.0156526

**Published:** 2016-06-02

**Authors:** Paula Tuzón, Jordi Solbes

**Affiliations:** Science Education Department, Facultat de Magisteri, Universitat de València, 46022 València, Spain; Universidad Veracruzana, MEXICO

## Abstract

The science learning process improves when the contents are connected to students’ lives. Particle physics has had a great impact in our society in the last years and has changed the theoretical picture about matter fundamental dynamics. Thus, we think that academic contents about matter components and interactions should be updated. With this study we aim to characterize the level of knowledge of high school students about this topic. We built a test with questions about classical atomic models, particle physics, recent discoveries, social implications and students opinions about it. Contrary to our first suspicion, students’ answers show a high variability. They have new physics ideas and show a great interest towards modern concepts. We suggest including an updated view of this topic as part of the curriculum.

## Introduction

Physics and Chemistry (P&Ch) Spanish Curriculum along the different secondary school levels is organized around topics related to the structure and interactions of matter. Motion dynamics, macroscopic forces, energy and work, gravitational and electrical fields, atoms, elements, molecules, chemical bonds, material properties and so on, are all concepts that try to develop the very first ideas about the basic components of matter and their interactions. Some of these concepts are usually introduced by using history of science, teaching different models that had been modified and changed while science and technology were moving forward. This methodology approaches students to the nature of science and contextualizes the steps performed in scientific progress [1].

However it is surprising that in the case of concepts related to matter components, this approach usually stops at the end of the 19th century or, luckily, at the beginning of the 20th century, ignoring the current picture provided by the Standard Model of Particle Physics [2, 3]. Moreover, modern concepts, when not ignored, appear disaggregated from the teaching sequence, isolated and incomplete, which brings even more confusion [3]. The aim of a secondary school level cannot be to provide a deep understanding of all complex new physics ideas, but to introduce some of them. There is no curricular or educational argument against it. On the contrary, studies suggest that presenting updated physics concepts improves learning and increases the interest towards science [4–9]. The Spanish P&Ch Curriculum includes concepts such as “atomic models”, “matter components” or “fundamental interactions”, but it does not detail either the content or “until when” (in history) students should learn those concepts. So, this historical bias is striking, especially given that one of the main recommendations included in the law describing this curriculum is “to introduce all the concepts following the nature of science and connecting the concepts with today’s society”.

The nature of science refers to the importance of learning procedural skills in science class, besides concepts. Among many procedural skills to develop in science education, there is the hypothesis or model building [10]. Historically, atomic and nuclear models about fundamental particles and their interactions have been built by refining the previous ones. Inquiry based science education studies [8, 11–13] strongly recommend to bring such refining process to science class. Models never represent closed truths, they always open new prospects when their limitations are analyzed. Under this point of view, it makes even less sense to stop the learning process on the 19th century atomic and nuclear models, because their prospects and limitations have already been overcome by other models along the 20th and 21st centuries. Avoiding these new models introduces an artificial and unjustified bias in the atomic and nuclear modelling process [8]. Moreover, this break on the models of matter timeline is sometimes faked [3], since, for example, neutrons use to be introduced at the same time as protons and electrons as part of the same model. Neutrons appeared later and only after the proton-electron model revision. Their role is therefore poorely understood.

By last, it is important to stress about the need to introduce updated concepts according to their obvious connection with the social context [14]. It is undeniable the impact that modern physics concepts of matter has had in our lives. The Standard Model of Particle Physics is behind most of the daily used new technology, the development of knowledge areas like Materials Science and Computer Science (e.g. Grid) or some new therapies and tools in medicine. Thus students might be interested about the modern physics behind the things that surround them. And precisely because of this contact, they probably have some (preliminary) conceptions about modern physics already, also coming from media, literature or films, which motivates a systematic treatment in class.

There are studies about students knowledge and misconceptions about the basic theories of modern physics, quantum mechanics and relativity [9, 15, 16]. However, data about the students knowledge of particle physics is scarce [6, 7, 9, 17] and further studies are needed. Information about students’ previous ideas and attitudes is essential in order to develop teaching strategies and interventions. The aim of this paper is to identify the lacks in comprehension and learning structure about matter components and their interactions towards their updated version. Do students know why there is matter and what is made of? Do they have an old-fashioned view of it? Do updated ideas show up in some way? Are they connected with curricula items?

## Materials and Methods

We developed a diagnostic test with free response questions about the knowledge and attitudes of students from the second-to-last year of high school, where the subject of P&Ch is included for the last time in the curriculum. Six groups (classes) of students from four high schools around Valencia metropolitan area (Spain) were surveyed. Students, teachers and high school principals provided oral consent for the development of this study. Student participation was voluntary. All the tests were anonymous and we did not collect any personal or identifying information from participants. Students were grouped by the high schools according to their elective (students can choose between P&Ch and Biology or P&Ch and Technical Drawing), when there are not enough students both kind of students were grouped together. A total of 138 students answered the test in January 2014, after covering most of the topics regarding the atom structure and interactions (the second semester of the course is devoted to study chemical bonds, materials’ properties and other matter topics at molecular level). Textbooks used by all these students do not present an updated view of the atomic structure, and only few modern concepts such as the strong and weak interactions are presented but marginally and decontextualized [3]. Group, high school, gender and elective were registered for each student. The test was composed by 17 questions divided into four main blocks:

Block (1) about atomic structure and interactions: (a) *What is matter made of?*, (b) *Which are the fundamental forces in Nature?*, (c) *Which is the force that keeps the electron bounded to the atomic nucleus?* and (d) *What is a photon?*

Block (2) about the model beyond the classical atom based on proton-neutron-electron and the electric interaction: (a) *If protons have the same electric charge, why are they able to remain so close in the atomic nucleus without repelling?*, (b) *What kind of interaction occurs when a nucleus transforms into another?* and (c) *Which particles do you think have been discovered?*

Block (3) about particle accelerators, colliders and current research: (a) *What is the Higgs boson?*, (b) *Have you heard of neutrinos? Do you know what they are?*, (c) *What is antimatter?*, (d) *Do you think it is dangerous? Why?*, (e) *What do you think that happens when two particles collide?* and (f) *What is the CERN?*

Block (4) about social connections: (a) *What is the use of colliding particles inside an accelerator?*, (b) *Why do you think it is important the work done in a particle physics centre?*, (c) *Do you think it has any impact in your daily life? Which one?* and (d) *Do you think there are enough contents about particle physics in high school classes? Would you like it to be otherwise? Why?*

The first block of questions aims to assess what students know about the classical model of the atom according to what is specified in the curricula. Block (2) tests students preconceptions about classical model limitations or new ideas and, implicitly, if a complete modelling technique has been performed in science class. Questions of this block might have been introduced at different points in the curricula and they appear in many of the books from the most widely distributed publishers [3]. Block (3) evaluates the use of the context in science class. These questions cover topical issues that students might have heard on the news or from other extra academic sources. Finally, Block (4) assesses students’ perceptions and interests towards particle physics.

The test was refined in a pilot study with 40 students from another public high school in Valencia metro. Redundant (Cramer’s V > 0.7) [18] or misleading questions were dropped or rephrased.

Answers to the questions (see S1 File) were classified into categories and each category was scored as low level (0 points), mid level (0.5 points) or high level (1 point) based on the expected level of knowledge that students are supposed to have according to the official P&Ch curriculum. Thus, *low level* reflects that the answer is below the expected level. *Mid level* means that the answer is at the level of classical models, i.e. in the answer there is no mention about any of the contributions that particle physics has made since the first half of the 20th century, so the answer is limited to the atomic model based on electrons, protons and neutrons and electromagnetic and gravitational interactions (no nuclear interactions, no subatomic particles). Finally, *high level* means that the answer shows (right or approximately right) ideas of updated concepts according to particle physics, beyond classical models.

The reliability of the test was measured with the Cronbach’s alpha [19] and the correlation among questions was assessed with the Cramer’s V. Differences in the total scores over the 17 questions were analyzed in terms of the registered variables: high school, group, elective and gender. This was done with a robust ANOVA [20, 21]. Only the effect of the main factors was explored (i.e, no interactions were included) as this was the model with the highest Akaike weight (i.e. the most probable model among all possible models) [22]. Total scores for each block of questions were also tested using the same methodology. A Holm’s correction [23] for multiple comparison was applied. All analyses were done with the statistical software R v.3.0.2 [24].

## Results and Discussion

The description of the sample according to gender and elective for the six groups of students is found in Table 1. The questionnaire presents a Cronbach’s alpha of 0.72 and a low redundancy among questions (Cramer’s V ∈ [0.24, 0.40]).

**Table 1:** Summary of the sample size (N) and sample distribution according to group, high school, gender and elective, where *Bio* means Biology and *Tech* means Technical Drawing, for each group of students.

			Gender (%)		Elective (%)	
Group	High School	N	Men	Women	Bio	Tech
1	C	16	68.75	31.25	0.00	100.00
2	C	20	40.00	60.00	100.00	0.00
3	D	23	52.17	47.83	60.87	39.13
4	B	37	72.97	27.03	64.86	35.14
5	A	23	30.43	69.57	73.91	26.09
6	A	19	73.68	26.32	0.00	100.00
Total		138	57.25	42.75	54.35	45.65

**Fig 1:**
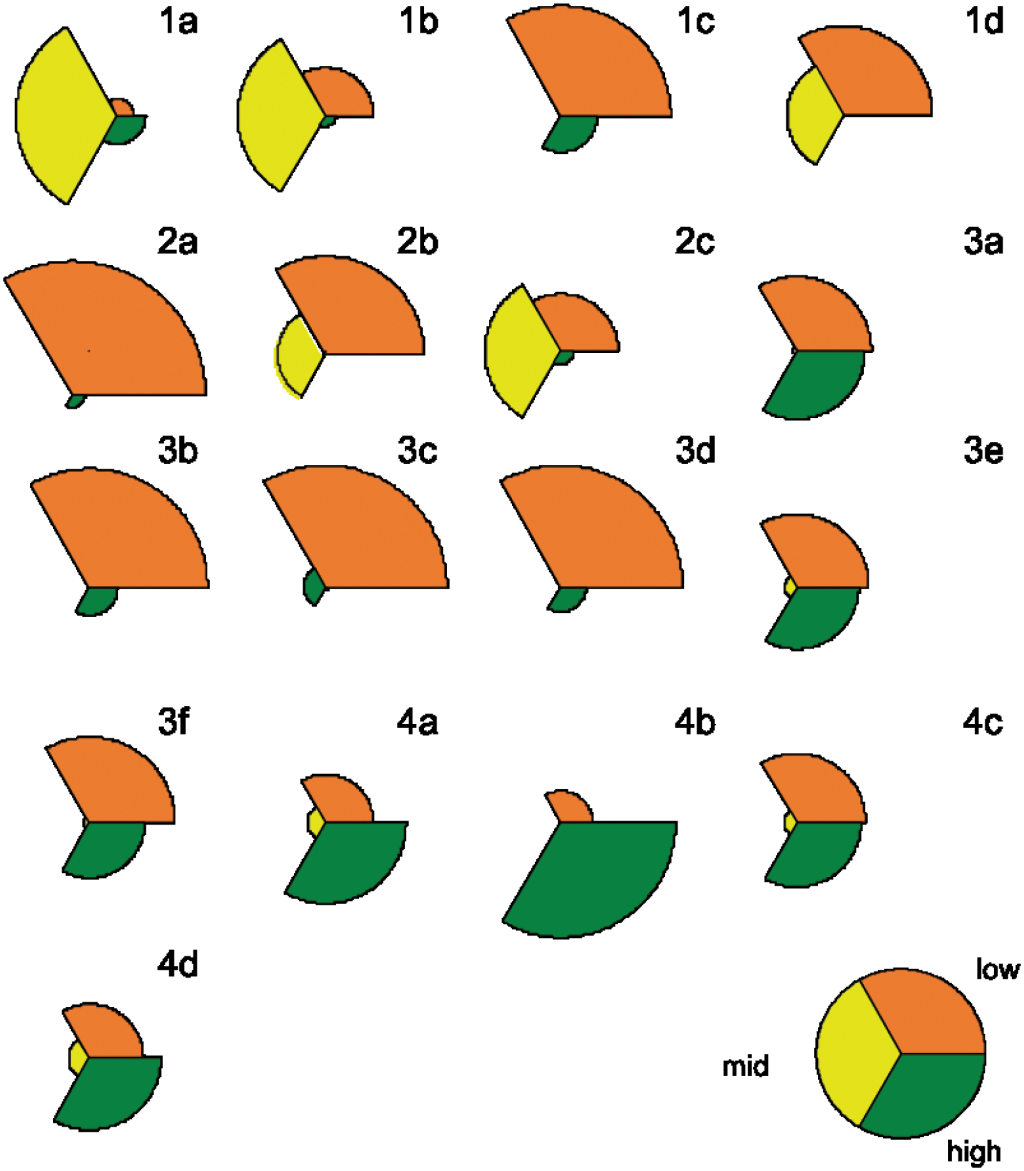
Global results for each of the 17 questions. Each colour stands for a score (orange, yellow and green are 0, 0.5 and 1 respectively). Radius length of each sector is proportional to its frequency.

Fig 1 shows the scores of the students’ answers to the test (see S1 File for complete answers). There is a high variability among questions.

In general, the level of students in Block (1) is intermediate (yellow), but for question (1c), *Which is the force that keeps the electron bounded to the atomic nucleus?* On average, students’ knowledge corresponds to the classical atomic models although the amount of answers scoring high is not negligible. This reflects that students have some ideas about current particle physics concepts. The poor results of the question (1c) are surprising. Only 22% of students provide a right answer; the most common erroneous answers refer to interactions like gravity (12%) or “the attraction force” (15%). These results show an obvious confusion regarding the types of interactions and the lack of hierarchy between fundamental (gravity, electromagnetic, strong and weak) and derived forces (all others). And also, they suggest that a common approach like the use of a planetary model to explain the atom as an electric system should be revised. Interestingly, “gravity” is the most common answer in question (1b). The level of students’ knowledge about Block (2) is mid-low. Almost none of the answers was scored as high. Question (2a) *If protons have the same electric charge, why are they able to remain so close in the atomic nucleus without repelling?* is the one where students perform worst across the whole test. On the one hand, this result was expectable judging for the information available in high school textbooks [3]; and on the other hand it is showing that in general the classical models limitations are not fully explored in the classroom, nor the questions or the new prospects that these limitations open. In other words, modelling as a constructive teaching strategy seems absent. However, despite students’ answers to question (2a) were mainly incorrect, they reflect creative misconceptions susceptible of being treated on a teaching intervention strategy within particle physics concepts. For example, many of them think that neutrons “neutralize” the electric repulsion from proton to proton, either by “absorbing” or “shielding” charge. They also talk about neutrons as “things to put between” protons, increasing the distance between them and so decreasing the effect of the electrical interaction. So, students think that neutrons must play a role here, which is true, but need to understand exactly how and why their hypotheses are wrong. A significant part (25%) of answers to (2b) talk about nuclear interaction or nuclear processes (proton/neutron interchange) but no one mentions the weak interaction. To cover these topics, the need of a strong force to solve question (2a) must be discussed. Then, the spontaneous decay of nucleons and the interplay between the strong and weak interactions could be introduced to explain unstable nuclei and could be also used to introduce neutrinos. Block (3) is the block of questions with the worst results. This is not surprising as the topics covered by these questions are not included in the Spanish P&Ch curriculum for the course. But at the same time, this is showing how science concepts are not connected to the social context in science class, since these questions have had a significant diffusion on media over the last years. It is noteworthy that, for example, 40% of students know that the Higgs is a fundamental particle related to the mass of the particles. Some students even go beyond that and connect mass with weight/gravity (e.g., “it is the particle responsible of the gravitational interaction”). Such considerations could be treated when introducing the interactions, in particular, when talking about gravity. There are also interesting answers like “it is a vacuum with mass” or “it is what remains when matter is removed from the particles”. These concerns should be clarified with easy models. 80% of students do not know what antimatter is and 50% think it is dangerous, either by “some sort of interaction with matter” or “due to black holes”. Antimatter, danger, black holes and swallowing are concepts somehow related for students, probably due to the influence of science fiction films or books (e.g., Dan Brown’s *Angels and Deamons*). However, not everything leads to destruction, one of the students concluded “if nothing has happened up to now and antimatter is part of Nature, it should not be dangerous”. This combination of concepts, misconceptions, and arguments should be taken into consideration in order to create an effective communication with society. Half of students know that new particles can be observed/created from collisions (question 3e). What are those particles? How many of them can be observed? Why? What for? All these questions naturally appear. Interestingly, some answers across the test show that at least some students get confused with atomic concepts and scales: “the atoms of the particles” or “the atoms of the nuclei”. This points out that a teaching intervention strategy is needed. Finally, the level of knowledge of Block (4) is somehow high, showing that students understand some of the implications of particle physics and emphasize their interest in modern physics. For example, in question (4b), 50% of students talk about discovering new particles with possible applications or highlight the great impact that this research has in our society. In question (4c) 40% of students identify such applications either with technology or with the knowledge about us and our world. Finally, most of the students express their interest about including this kind of physics (question (4d)) as part of the curriculum in different ways.

Variability among students also exists, and statistically significant differences due to gender (p-value = 0.042) or high school (p-value = 0.004) were found when considering students’ scores for the whole test. In the case of gender, these differences appear mainly in Block (3) (p-value = 0.001) that is related to concepts that go beyond what is covered by the course curriculum. Men scored better in this block. Interestingly, blocks of questions more directly related to academic contents (Blocks (1) and (2)) are the ones that show significant or marginally significant differences between high schools (p-values 0.002 and 0.093 respectively). Further study will be needed to test if these differences are due to different teaching methodologies or to other variables not explored in this study as students’ grades or socio/economic factors.

Contrary to what could be expected, students do have knowledge about particle physics despite not being covered by the traditional curricula. However, this knowledge is partial, unstructured and there is a great variability among students and topics, which motivates the need of a teaching intervention strategy consistent with modelling techniques. The idea is not to add an extra item in the high school curriculum about Particle Physics, but to encourage teachers to lead the discussions about matter components and interactions beyond the 19th century until nowadays [25]. Most of these questions can easily fit into the current curricula. For example, when studying atomic models, question (2b) should trigger the discussion towards the need of the strong interaction and new particles (quarks) that can feel it and compose the protons. This also clarifies the role of the neutrons in the nucleus. The weak interaction explains spontaneous proton/neutron decays that easily connect with the stability of the atomic nucleus and radioactivity. When talking about forces, the four fundamental interactions and their role/interplay in the formation of matter should be treated. Later in the course, when macroscopic forces like friction or the elastic force appear, it is important to recall that all these forces can be understood in terms of the fundamental ones; and that almost all macroscopic forces have an electric origin. Transverse to all topics, it is important to search news about current searches and applications of particle physics, which can be used to engage students in debates about concepts like antimatter or the Higgs boson, for example, and to clarify controversies like neutrino’s speed or LHC’s dangers. Further investigation is needed to provide effective teaching interventions that could help to bridge the gap from the 19th century physics taught in High Schools to modern and up-to-date ideas of Nature.

## Conclusions

In this paper, the level of knowledge about the structure and interactions of matter has been evaluated, according to whether or not this knowledge shows an updated view of the topic. The knowledge of students adjusts globally to a mid-low level, i.e., to the classical models. However, the variability of answers is high. Ideas from new models appear, meaning that students somehow know updated concepts. Nonetheless, these ideas are very tentative and show confusions with both new and classical models. Our results show that students are highly interested towards particle physics and they are curious about the social implications of the topic. The whole picture justifies the need of a teaching intervention strategy to integrate the new concepts in the learning process; so that the classical models can be correctly understood and the topic about matter turns out to be unbiased and completed. Given the social impact of modern physics, this necessity is reinforced. However, how this can be done in an effective manner has been barely investigated (see [9] and references therein). Our goal for a further study is to present a teaching intervention strategy. It is based on interactive engagement [26] and modelling techniques with embodiment [27]. Using *embodiment*, students perform as the active agents of the model, which greatly facilitates the understanding and learning process.

## Supporting Information

S1 File Categories for students’ answers to the questionnaire. Tables 1, 2, 3 and 4 describe the answers categories for Block (1), (2), (3) and (4) of questions from the pretest. The tables show, for each question referenced on the first column, the different types of answers considered as categories (second column), their corresponding score (third column) and the percentage of students answers that fit into that category (fourth column).
(PDF)

S2 File Questionnaire. Blank copy of the questionnaire given to students.
(PDF)
