# Supplementary material for: Particle Physics in High School: A Diagnose Study
Source: PLoS One. 2016 Jun 2;11(6):e0156526. doi: 10.1371/journal.pone.0156526 (PMC4890794; doi:10.1371/journal.pone.0156526)
Supplement: S1 File — Tables 1, 2, 3 and 4 describe the answers categories for Block (1), (2), (3) and (4) of questions from the pretest. The tables show, for each question referenced on the first column, the different types of answers considered as categories (second column), their corresponding score (third column) and the percentage of students answers that fit into that category (fourth column). (PDF) [file pone.0156526.s001.pdf]

S1 File

Find bellow the Tables 1, 2, 3 and 4 describing the answers categories for Block (1), (2), (3) and (4) of questions from the pretest. The tables show, for each question referenced on the first column, the different types of answers considered as categories (second column), their corresponding score (third column) and the percentage of students answers that fit into that category (fourth column).

Table 1: **Answers categories for Block (1) of questions.**

Question	Answer category	Score	%
1a	Atoms and particles	1	18.12
	Atoms	0.5	68.84
	Others	0	11.59
	NA	0	1.45
1b	Gravity, electromagnetic, strong and weak	1	6.52
	Gravity	0.5	21.74
	Gravity and others	0.5	22.46
	Electromagnetic and others	0.5	2.90
	Gravity and electromagnetic	0.5	11.59
	Gravity, nuclear and others	0.5	1.45
	Attraction	0	3.62
	Others	0	19.57
	NA	0	10.14
1c	Electric or electromagnetic	1	22.46
	Attraction	0	15.22
	Magnetic	0	11.59
	Gravity	0	11.59
	Others	0	26.09
	NA	0	13.04
1d	Electromagnetic particle/wave	1	0.72
	Particle of light	0.5	26.09
	Wave/particle of light	0.5	2.90
	Radiation or energy box that the electron gets/emits when changes orbit	0.5	5.80
	Something of light	0	12.32
	Others	0	17.39
	NA	0	34.78

Table 2: **Answers categories for Block (2) of questions.**

Question	Answer category	Score	%
2a	Gluons or other force stronger than the electric force	1	8.70
	Protons charge cancels with electrons charge	0	9.42
	Neutrons neutralize protons charge or separates them	0	15.22
	Because of the neutrons (*don't say why*)	0	25.36
	Others	0	23.91
	NA	0	17.39
2b	Nuclear	0.5	2.90
	Fusion/fission	0.5	13.04
	Gives/gets protons or neutrons (change of element)	0.5	10.87
	Radioactive	0.5	5.80
	Gives/gets electrons	0	6.52
	Others	0	17.39
	NA	0	43.48
2c	Many (*and cite more than two of the categories below*)	1	7.97
	Higgs	0.5	21.01
	Electron, proton and neutron	0.5	25.36
	Neutrinos	0.5	0.72
	Quarks	0.5	2.90
	Many (*and don't mention any*)	0	18.12
	Others	0	6.52
	NA	0	17.39

Table 3: **Answers categories for Block (3) of questions.**

Question	Answer category	Score	%
3a	Elementary particle (*and mention a property*)	1	31.16
	Provides mass/matter	1	10.87
	Creates eveything	0.5	3.62
	Force	0	0.72
	God's particle	0	3.62
	Others	0	22.46
	NA	0	27.54
3b	Yes, particles (*and mention a property: small, fast...*)	1	14.49
	Yes, without charge	1	2.90
	No, no	0	23.91
	Yes, no	0	24.64
	Yes, faster than light	0	0.72
	Yes, inside the neutrons or something of neutrons	0	8.70
	Others	0	9.42
	NA	0	15.22
3c	Matter with opposite charges	1	2.17
	Swallows or destroys matter (releasing energy)	0.5	13.77
	Something of black holes	0	2.17
	Some tautology like "all that is not matter"	0	29.71
	Others	0	32.61
	NA	0	19.57
3d	Yes, releases energy when finds matter or destroys it	1	15.94
	Yes (*don't say why*)	0	12.32
	Yes, something of black holes or something absorbing/swallowing matter	0	5.80
	No	0	18.84
	Others (*think it is dangerous for other reasons*)	0	18.84
	NA	0	28.26
3e	Other particles are created	1	14.49
	They break	1	25.36
	Explosion	0.5	7.97
	Fusion of particles	0	10.14
	Fusion of nuclei or something of nuclei	0	2.90
	Energy release	0	17.39
	Others (*many say they rebound*)	0	13.77
	NA	0	7.97
3f	Research center (and something about the accelerator)	1	34.06
	Related to the accelerator	0.5	3.62
	Accelerator	0	16.67
	Others	0	8.70
	NA	0	36.96

Table 4: **Answers categories for Block (4) of questions.**

Question	Answer category	Score	%
4a	To know particle properties	1	29.71
	To discover new particles	1	23.19
	To recreate states or situations	0.5	4.35
	To know about the origin of the Universe and the Big-Bang	0.5	3.62
	To break them	0.5	3.62
	Others	0	26.81
	NA	0	8.70
4b	Know the Universe	1	10.14
	Discover new things/particles (and possible applications)	1	26.81
	Find out what we are made of	1	14.49
	Importance of research in our society	1	23.19
	Get energy	0	4.35
	Others	0	3.62
	NA	0	17.39
4c	Yes, understand the world and what we are made of	1	13.77
	Yes, technology and lifestyle	1	26.81
	Yes, but not now, in the future	0.5	5.80
	Yes, but not directly	0.5	2.90
	No	0	18.84
	Yes (*don't say which one*)	0	8.70
	Others	0	3.62
	NA	0	19.57
4d	No and I would like out of curiosity and because we alwaysa do the same physics	1	50.00
	No and I would like but not in P&Ch (*refer to other transversal subjects like "culture and science"*)	0.5	0.72
	No and I would like but not evaluable	0.5	2.17
	No and I would like but as an elective	0.5	0.72
	No and it is not possible because we already have enough material to study	0.5	2.17
	No and it is not possible because of admission tests to college	0.5	0.72
	No and yes but it is a difficult topic	0.5	7.25
	Yes, there are enough	0	16.67
	No and that's right	0	10.14
	NA	0	9.42
