# Supplementary material for: Particle Physics in High School: A Diagnose Study
Source: PLoS One. 2016 Jun 2;11(6):e0156526. doi: 10.1371/journal.pone.0156526 (PMC4890794; doi:10.1371/journal.pone.0156526)
Supplement: S2 File — Blank copy of the questionnaire given to students. (PDF) [file pone.0156526.s002.pdf]

Gender: High School:

Elective:

This questionnaire expects you to express your knowledge about the topic, not only based on what you learned in class, but also from media (TV, Internet...).

1.- What is matter made of?

2.- Which are the fundamental forces in Nature?

3.- Which is the force that keeps the electron bounded to the atomic nucleus?

4.- If protons have the same electric charge, why are they able to remain so close in the atomic nucleus without repelling?

5.- What kind of interaction occurs when a nucleus transforms into another?

6.- What is a photon?

7.- Which particles do you think have been discovered?

8.- What is the Higgs boson?

9.- Have you heard of neutrinos? Do you know what they are?

10.- What is antimatter?

11.- Do you think it is dangerous? Why?

12.- What do you think that happens when two particles collide?

13.- What is the CERN?

14.- What is the use of colliding particles inside an accelerator?

15.- Why do you think it is important the work done in a particle physics centre?

16.- Do you think it has any impact in your daily life? Which one?

17.- Do you think there are enough contents about particle physics in high school classes? Would you like it to be otherwise? Why?
